# Heterologous production of fungal secondary metabolites in Aspergilli

**DOI:** 10.3389/fmicb.2015.00077

**Published:** 2015-02-10

**Authors:** Diana Chinyere Anyaogu, Uffe Hasbro Mortensen

**Affiliations:** Section for Eukaryotic Biotechnology, Department of Systems Biology, Technical University of DenmarkKongens Lyngby, Denmark

**Keywords:** secondary metabolite, *Aspergillus*, gene clusters, fungi, heterologous expression, polyketide synthase

## Abstract

Fungal natural products comprise a wide range of compounds. Some are medically attractive as drugs and drug leads, some are used as food additives, while others are harmful mycotoxins. In recent years the genome sequence of several fungi has become available providing genetic information of a large number of putative biosynthetic pathways. However, compound discovery is difficult as the genes required for the production of the compounds often are silent or barely expressed under laboratory conditions. Furthermore, the lack of available tools for genetic manipulation of most fungal species hinders pathway discovery. Heterologous expression of the biosynthetic pathway in model systems or cell factories facilitates product discovery, elucidation, and production. This review summarizes the recent strategies for heterologous expression of fungal biosynthetic pathways in Aspergilli.

## INTRODUCTION

Filamentous fungi produce a plethora of secondary metabolites, SMs, like polyketides (PK), terpenes, and non-ribosomal peptides (NRP). Several fungal SMs dramatically impact human life either because they are harmful mycotoxins, like carcinogenic aflatoxin (Eaton and Gallagher, 1994) and fumonisin (Voss and Riley, 2013), or because they are used to efficiently combat human disease, e.g., penicillin and lovastatin (Campbell and Vederas, 2010). Importantly, analyses of fully sequenced fungi show that the number of SMs known to be produced by these fungi is too low to account for the number of genes and gene clusters that potentially may lead to production of SMs (Szewczyk et al., 2008). This strongly suggests that the chemical diversity of the metabolomes produced by filamentous fungi is much larger than what is currently known, and it is therefore very likely that new harmful mycotoxins and new blockbuster drugs await discovery.

The rapid accumulation of fully sequenced genomes has accelerated the discovery of novel SMs dramatically. However, this sequence resource cannot be directly translated into chemical structures of new compounds despite that genes and gene clusters are often readily identified by bioinformatics tools (Khaldi et al., 2010; Andersen et al., 2013; Blin et al., 2013). For example, the exact structures of products released by fungal type I polyketide synthases are difficult to predict due to the iterative use of the different catalytic domains in these enzymes. Similarly, subsequent decorations performed by tailoring enzymes encoded by other genes in the cluster toward formation of the mature end product(s) are complex and not easy to predict. Another challenge is that many SMs are not readily produced under laboratory conditions although several approaches have been successfully employed to activate silent clusters (for reviews, see Brakhage and Schroeckh, 2011; Chiang et al., 2011; Klejnstrup et al., 2012; Wiemann and Keller, 2014; Yaegashi et al., 2014). To link novel SMs to genes, and to map novel biosynthetic pathways, extensive genetic manipulations of the strains are typically required. Since, most new gene clusters uncovered by sequencing projects will be situated in fungi with no available genetic tools, this type of analysis may not be straight forward. Moreover, it may be difficult to purify sufficient amounts of a desired compound from these fungi to allow for thorough characterization of its bioactivity. An alternative approach is to transfer genes and gene clusters to hosts with strong genetic toolboxes thereby facilitating product discovery, production, and characterization. Here we review SM production in Aspergilli based cell factories. Considerations and strategies concerning central steps toward fungal SM production are presented for inspiration: choice of host, how to produce the first intermediate in the pathway, and how to establish the remaining part of the pathway.

## HOST CHOICE FOR HETEROLOGOUS EXPRESSION OF FUNGAL SECONDARY METABOLITES

Heterologous expression of SM genes has mainly been performed in baker’s yeast *Saccharomyces cerevisiae* (Tsunematsu et al., 2013) and in the filamentous fungi *Aspergillus oryzae* and *A. nidulans*. Each of these model organisms offers specific advantages. For *S. cerevisiae* a superior genetic toolbox for strain construction has been developed and novel genes can easily be engineered into a wealth of single- and multi-copy expression plasmids or into chromosomes. For example, gene targeting and fusion of DNA fragments by homologous recombination (HR) is highly efficient in *S. cerevisiae*. Moreover, *S. cerevisiae* contains an insignificant endogenous secondary metabolism (Siddiqui et al., 2012). This fact simplifies the analysis of strains equipped with new pathways as they are not complicated by the presence of a multitude of other SMs; and the risk of undesirable side reactions due to cross chemistry between the novel and endogenous pathways is minimized. However, lack of secondary metabolism also means that yeast is not naturally geared for SM production and may contain limiting amounts of, or even lack, relevant building blocks (Kealey et al., 1998; Mutka et al., 2006). Moreover, localization of relevant enzymes for aflatoxin production into specialized vesicles in *A. parasiticus* indicate that fungi may possess specialized compartments for SM production, which yeast may not contain (Roze et al., 2011); and as introns are few in *S. cerevisiae* (Spingola et al., 1999) and differ from those in filamentous fungi (Kupfer et al., 2004), mRNA splicing could be problematic. For these reasons filamentous fungi may often be more appropriate for heterologous SM production. *A. oryzae* is often used for this purpose because it possesses a limited endogenous secondary metabolism and *A. nidulans* because a strong genetic toolbox has been developed for this fungus (for review see, Meyer, 2008; Meyer et al., 2011). Importantly, the recent development of efficient tools for gene targeting in filamentous fungi, including strains where random integration is minimized due to mutation of genes required for non-homologous end-joining (Ninomiya et al., 2004; Nayak et al., 2006; Takahashi et al., 2006), has further stimulated the use of these organisms as hosts for SM pathway reconstitution experiments.

## HETEROLOGOUS EXPRESSION OF POLYKETIDE SYNTHASES

The fact that the product(s) released by fungal type I PK synthases (PKSs) cannot easily be predicted from their primary sequence has sparked a major interest in expressing PKS genes in model fungi with the aim of identifying these products. In yeast, two 2 μ based multi-copy plasmids harboring the 6-methylsalicylic acid (6-MSA) synthase gene from *Penicillium patulum* and the PKS activating PPTase gene from *Bacillus subtilis* were successfully used to produce 6-MSA (Kealey et al., 1998). Similarly production of green pigment has been achieved in a *wAΔ yAΔ* (white) *A. nidulans* (Holm, 2013) via co-expression of the PKS gene *wA* and laccase gene *yA* harbored on two AMA1 (Aleksenko and Clutterbuck, 1997) based plasmid. However, if multiple plasmids are needed to form a complex end-product, these vectors may have limited value since sufficient markers may not be available, and since 2 μ and AMA1 plasmids segregate unevenly during mitosis (Albertsen et al., 2011; Holm, 2013; Jensen et al., 2014).

More stable expression has been achieved by integrating PKS genes randomly into the genome of a model filamentous fungus via the non-homologous end-joining pathway. Using this concept, Fujii et al. (1996) successfully linked 6-MSA production to the PKS gene *atX* from *A. terreus* by expressing *atX* host *A. nidulans.* Considering that foreign SMs may be toxic in the new host, it is advisable to employ an expression strategy that minimizes this risk. For production of 6-MSA and enniatins in *A. nidulans* and *A. niger*, this was achieved by fusing the PKS and NRPS genes to the inducible promoters, amyB (Fujii et al., 1996) and Tet-on (Richter et al., 2014), respectively. Over the years, a number of other PKS genes have been linked to products using this strategy in *A. nidulans* and *A. oryzae* including the PKS genes for production of 1,3,6,8-tetrahydroxynaphthalene, alternapyrone, and 3-methylorcinaldehyde by (Fujii et al., 1999, 2005; Bailey et al., 2007).

Random integration may trigger unpredictable pleiotropic effects that alter the expression of neighboring genes, hence, complicating subsequent analyses (Verdoes et al., 1995; Palmer and Keller, 2010). Moreover, since multiple copies of the gene often integrate simultaneously into the same site, strains may suffer genetic instability and lose expression over time. Taking advantage of the development of strains and techniques for efficient gene targeting, these problems can be eliminated by inserting genes into a defined locus. This facilitates not only subsequent strain characterization, but also sets the stage for experiments analyzing mutant varieties of the gene where equal expression levels of the alleles are important to fairly judge the impact of individual mutations. Using this approach, Hansen et al. (2011) demonstrated that *mpaC* from *P. brevicompactum* encodes a PKS producing 5-methylorsellinic acid. In this case, *mpaC* was introduced into a defined site, *IS1*, on chromosome I of *A. nidulans*, which supports expression of non-toxic genes in a variety of tissues without affecting fitness. Moreover, to simplify the integration of genes into *IS1*, a set of vectors pre-equipped with targeting sequences, genetic markers, promoters and terminators and a USER-cloning cassette (Nour-Eldin et al., 2006) allowing for seamless ligation free insertion of relevant genes into the vector was developed. Using this technology, *ausA*, from *A. nidulans*, and *yanA*, from *A. niger*, have been shown to encode PKSs producing 3-,5-dimethyl orsellinic acid and 6-MSA, respectively (Nielsen et al., 2011; Holm et al., 2014). In a variation of this approach, Chiang et al. (2013) used fusion Polymerase chain reaction (PCR) to merge an *alcA* promoter and PKS genes followed by integration into the *wA* locus of *A. nidulans*. Correctly targeted transformants could therefore easily be identified as white colonies. The authors expressed nine non-reducing (NR) PKS genes from *A. terrreus* in this manner and identified six products. Heterologous production of PKs is complicated by the fact that not all synthases possess a domain providing a product release mechanism (Awakawa et al., 2009; Du and Lou, 2010) and by the fact that some PKSs require a starter unit different from Ac-CoA (Hoffmeister and Keller, 2007). In the study by Chiang et al. (2013), two of the nine NR-PKSs analyzed did not contain such a domain and for one, a product was achieved by co-expressing a gene encoding a thioesterase activity. In addition, two NR-PKS were predicted to employ unusual starter units. For one NR-PKS, production of this starter unit was successfully delivered by co-expressing a gene encoding a highly reducing PKS and the collaborative effort of the two enzymes resulted in production of an intermediate for production of asperfuranone (Chiang et al., 2013).

## TRANSFER OF GENE CLUSTERS TO HETEROLOGOUS HOSTS

**FIGURE 1:**
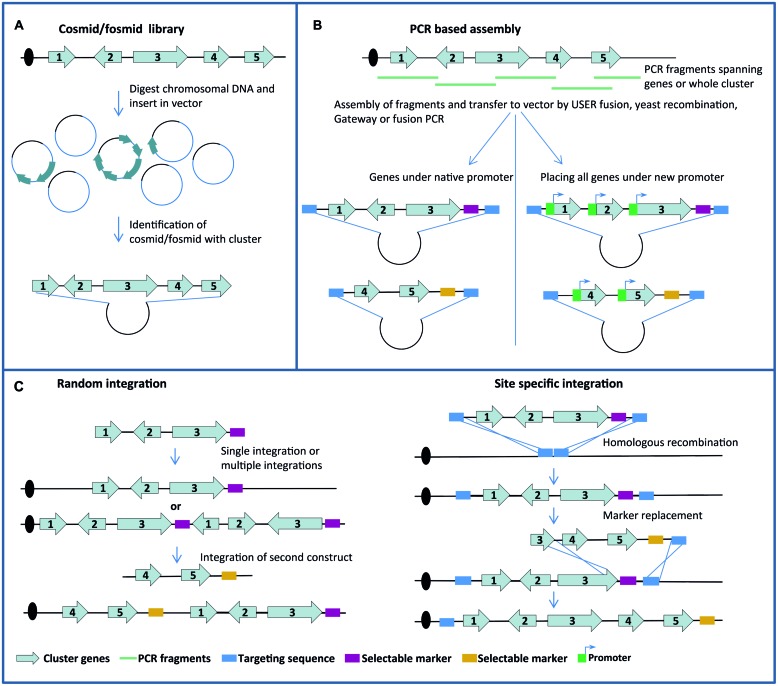
**Overview of principles employed for constructing DNA fragments **(A,B)** and for integration in host genomes (C)**.

Reconstitution of most SM pathways depends on the expression of multiple genes since the SM scaffold delivered by the synthase is further decorated by tailoring enzymes. Moreover, genes providing transcriptions factors, transporters and/or a resistance mechanism may also be required. Construction of strains for heterologous end-product production is therefore a major challenge as it requires not only transfer, but also activation, of large gene clusters. Two principles are generally employed for constructing DNA fragments that allow transfer of gene clusters into another fungal host. Firstly, DNA fragments harboring entire, or a large part of, gene clusters have been identified in cosmid/fosmid libraries and transferred into vectors with a selectable fungal marker (**Figure 1A**). Secondly, PCR fragments covering the gene cluster have been stitched together using a variety of methods including USER Fusion, Gateway cloning and yeast recombination to create suitable transformation vectors (**Figure 1B**). When gene clusters have been transformed into the host, activation has been achieved by three different methods. Firstly, in cases where the native gene cluster harbors a TF gene, it has been possible to activate the genes in the cluster by equipping the TF gene with a constitutive or inducible promoter known to work in the host. Secondly, in gene clusters without a TF gene, activation has been achieved either by overexpressing the global regulator LaeA or by individually swapping cluster gene promoters for constitutive or inducible promoters. Like for integration of PKS genes, and for the same reasons, integration strategies based on random or directed integration have been used (**Figure 1C**). In many cases these strategies have been combined and successful examples are provided below.

Cosmids harboring the entire penicillin biosynthetic pathway from *P. chrysogenum* were introduced to *Neurospora crassa* and *A. niger*, resulting in the production of penicillin (Smith et al., 1990). Similary, cosmids harboring the citrinin biosynthetic pathway from *Monascus purpureus* and the monacolin K gene cluster from *Monascus pilosus* were individually integrated into random positions in the genome of *A. oryzae*. In the case of citrinin, the transformant directly produced citrinin, but in small amounts. However, as the cluster contains a TF gene, additional copies of the activator gene (*ctnA*) controlled by the *A. nidulans trpC* promoter were subsequently introduced in the strain to boost production. Impressively, this resulted in a 400 fold increase of citrinin production (Sakai et al., 2008). The monacolin K gene cluster does not contain a TF gene. In this case, the cluster was activated by overexpressing the gene encoding the global activator LaeA (Sakai et al., 2012). A limitation of this strategy may be difficulties in isolating cosmids containing a fragment that harbors the entire gene cluster, especially if clusters are large. For example, the reconstruction of the terrequinone A gene cluster in *A. oryzae* was based on a fosmid containing an incomplete gene cluster. The remaining part of the cluster was subsequently obtained by PCR, cloned into a vector and transformed into the *A. oryzae* strain harboring the partial terrequinone A gene cluster (Sakai et al., 2012).

Several PCR based strategies have been used for transferring gene clusters from the natural producer to a model fungus. For clusters harboring a TF gene, PCR fragments covering the entire gene cluster have been amplified, fused, and inserted via a single cloning step into vectors predestined for site specific integration in the genome of the host by HR. Multiple PCR fragments can be orderly assembled by different strategies. For example, PCR fragments of the geodin and neosartoricin B clusters were physically linked by *E. coli* based USER fusion and by yeast based HR, respectively (Nielsen et al., 2013; Yin et al., 2013). Importantly, in both cases the promoter controlling expression of the TF gene was swapped for a strong constitutive promoter during the cluster re-assembly process. Large inserts (>15 kb) may not be propagated stably in a cloning vector and large clusters need to be subdivided into smaller fragment cassettes, which together represent the entire cluster. Multiple subsequent integrations depend on marker recycling, which can be achieved by using *pyrG* as a selectable/counterselectable marker. A faster method employs a two marker system for cluster transfer (Nielsen et al., 2013). During one transformation cycle, one of the markers is used to select for integration of the first cluster cassette and the other marker for the next cassette. By ensuring that integration of one cassette eliminates the marker contained by the preceding cassette, numerous cluster cassettes can be integrated sequentially by alternating the use of the two markers. Advantageously, when the gene clusters is inserted in a controlled manner it can be subjected to further genetic dissection to clarify the biochemical pathway toward end product. With the geodin cluster this was exploited to demonstrate that *gedL* encodes a halogenase using sulochrin as substrate (Nielsen et al., 2013).

Polymerase chain reaction based reconstruction of clusters that do not contain an activating TF gene requires more elaborate genetic engineering as all cluster genes need to be equipped with new promoters and terminators. In one strategy, cluster ORFs were inserted either individually or in pairs into expression cassettes in plasmids carrying different selection markers. Using this approach several small gene clusters containing four to five genes have been, fully or partially, reconstituted by randomly introducing the genes into the genome of *A.* oryzae. Several SMs have been achieved by this method including tennelin, pyripyropene, aphidicolin, terretonin, and andrastin A (Heneghan et al., 2010; Itoh et al., 2010; Fujii et al., 2011; Matsuda et al., 2012,^2013^). Construction of larger clusters in *A. oryzae* has been limited by the number of available markers. To bypass this problem, Tagami et al. (2013, 2014) used the high co-transformation frequency with *A. oryzae* to integrate two vectors in one round of transformation using selection for only one marker. This allowed for reconstituting clusters with six and seven genes for production of paxilline and aflatrem, respectively (Tagami et al., 2013, 2014). Addressing the same problem, Gateway cloning was used to construct expression vectors containing up to four genes (Pahirulzaman et al., 2012; Lazarus et al., 2014). Utilizing this approach Wasil et al. (2013) expressed different combinations of the synthase and tailoring genes from the aspyridone pathway from *A. nidulans* in *A. oryzae*. An alternative approach to save markers is to generate synthetic polycistronic genes where all genes in the construct are under the control of a single promoter and where all ORFs are separated by a sequence encoding the viral 2A peptide that results in co-translational cleavage, hence, resulting in the formation of independent enzymes (Kim et al., 2011). Using this concept Unkles et al. (2014) reconstituted the penicillin gene cluster from *P. chrysogenum* as a single three ORF polystronic gene by yeast mediated HR. Random genomic integration of this construct resulted in penicillin production in *A. nidulans*.

A strategy for gene cluster activation based on promoter/terminator swapping has also been implemented in gene cluster transfer methods where genes are inserted into defined integration sites (Hansen et al., 2012; Mikkelsen et al., 2012; Chiang et al., 2013). Specifically, expression plasmids containing one to two cluster genes were constructed by USER cloning or by fusion PCR and integrated into the expression sites in *S. cerevisiae* and *A. nidulans* to allow for production of the pigment precursor rubrofusarin in yeast (Rugbjerg et al., 2013) and for partial and fully reconstitution of the pathways for mycophenolic acid and asperfuranone production, respectively, in *A. nidulans* (Hansen et al., 2012; Chiang et al., 2013).

## PERSPECTIVES

The rapid development of molecular tools for cluster transfer and re-engineering in heterologous hosts is now at a stage where high-throughput experiments can be performed, and we therefore predict that novel SMs, genes, pathways and enzymes routinely will be discovered using this approach. For now most efforts have been proof of principle cases analyzing genes and gene clusters from genetically well-characterized organisms, but the next wave of breakthroughs will likely concern SMs originating from genetically exotic fungi. In addition, the natural reservoir of SMs will likely expand dramatically as synthetic biology based approaches using bio-bricks of promoters, terminators and SM genes are combined in intelligent or in random ways in model fungi to deliver compounds that nature never invented. Together, we envision that heterologous production will serve as a major driver for SM discovery and development delivering compounds that can be used in the food and pharma industries. Accordingly, physiologically well-characterized fungal cell factories should preferentially be employed as platforms for novel SMs discovery and development. These fungi display superior fermentation properties and extensive metabolic engineering toolboxes, hence, shortening the way toward large scale production.

## Conflict of Interest Statement

The authors declare that the research was conducted in the absence of any commercial or financial relationships that could be construed as a potential conflict of interest.
